# Supplementary material for: The Tissue Expression Divergence of the WUSCHEL-Related Homeobox Gene Family in the Evolution of Nelumbo
Source: Plants (Basel). 2025 Jun 21;14(13):1909. doi: 10.3390/plants14131909 (PMC12251702; doi:10.3390/plants14131909)
Supplement: Supplementary file 1 [file plants-14-01909-s001.zip › Figure S1-S3.pptx]

## Slide 1
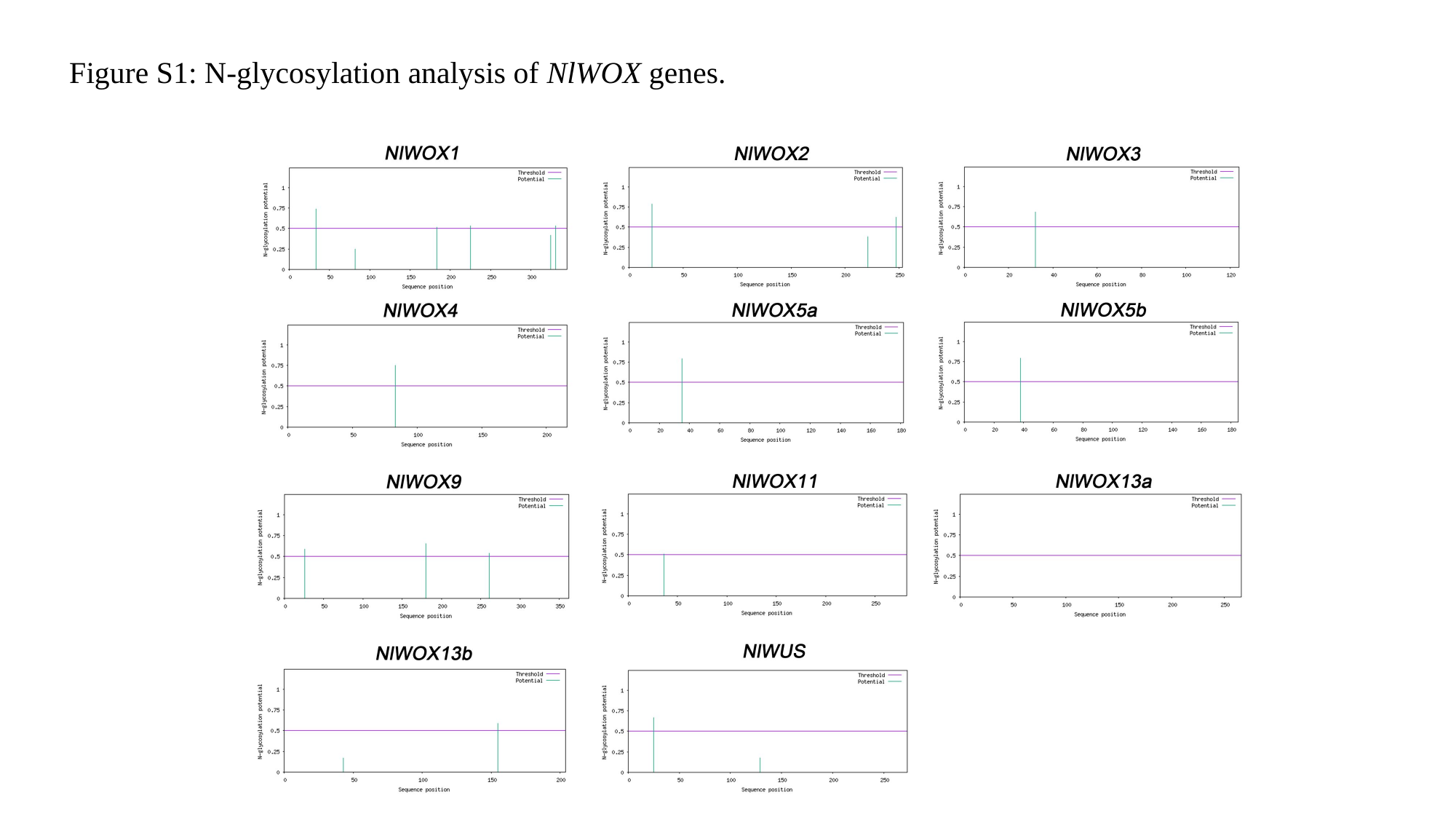

Figure S1: N-glycosylation analysis of NlWOX genes.

## Slide 2
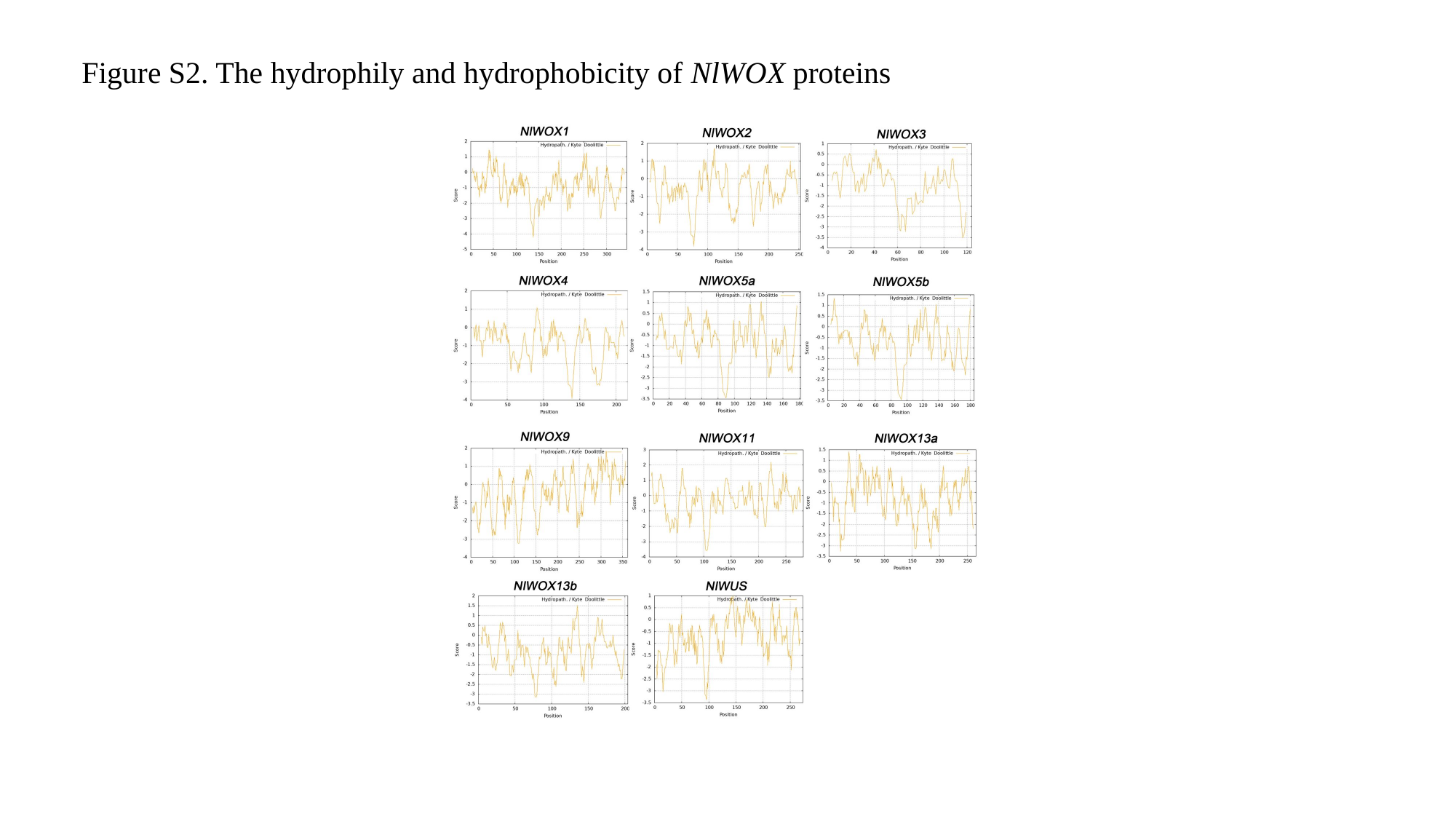

Figure S2. The hydrophily and hydrophobicity of NlWOX proteins

## Slide 3
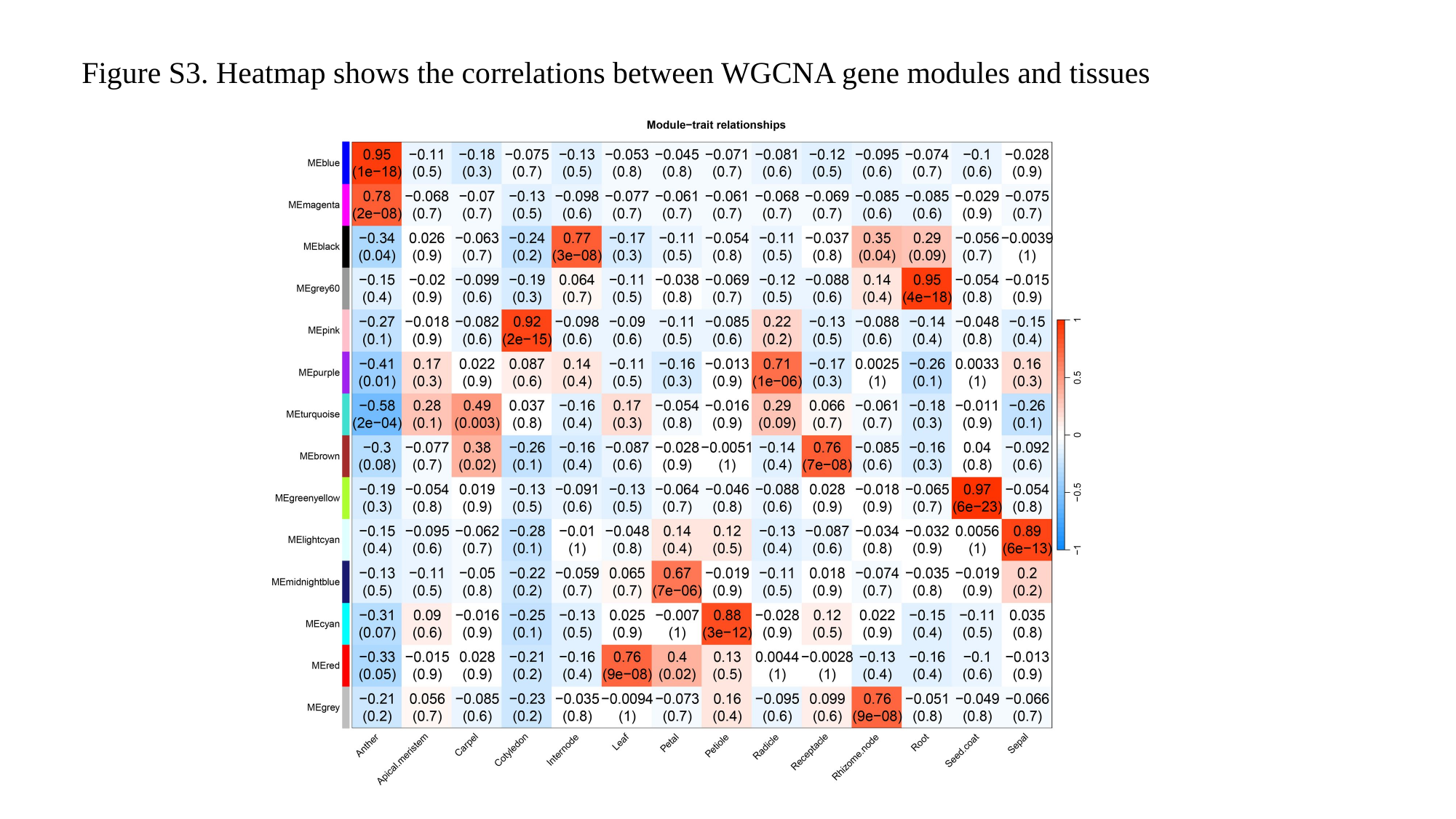

Figure S3. Heatmap shows the correlations between WGCNA gene modules and tissues
